# Parietal plasticity after training with a complex video game is associated with individual differences in improvements in an untrained working memory task

**DOI:** 10.3389/fnhum.2014.00169

**Published:** 2014-03-21

**Authors:** Aki Nikolaidis, Michelle W. Voss, Hyunkyu Lee, Loan T. K. Vo, Arthur F. Kramer

**Affiliations:** ^1^Neuroscience Program, University of Illinois, Urbana-ChampaignUrbana, IL, USA; ^2^Beckman Institute, University of Illinois Urbana-ChampaignUrbana, IL, USA; ^3^Department of Psychology, University of IowaIowa City, IA, USA; ^4^Brain Plasticity InstituteSan Francisco, CA, USA; ^5^Department of Electrical Engineering, Tan Tao UniversityLong An, Vietnam; ^6^Department of Psychology, University of Illinois Urbana-ChampaignUrbana, IL, USA

**Keywords:** cognitive training, neuroplasticity, tranfser, working memory, video games

## Abstract

Researchers have devoted considerable attention and resources to cognitive training, yet there have been few examinations of the relationship between individual differences in patterns of brain activity during the training task and training benefits on untrained tasks (i.e., transfer). While a predominant hypothesis suggests that training will transfer if there is training-induced plasticity in brain regions important for the untrained task, this theory lacks sufficient empirical support. To address this issue we investigated the relationship between individual differences in training-induced changes in brain activity during a cognitive training videogame, and whether those changes explained individual differences in the resulting changes in performance in untrained tasks. Forty-five young adults trained with a videogame that challenges working memory, attention, and motor control for 15 2-h sessions. Before and after training, all subjects received neuropsychological assessments targeting working memory, attention, and procedural learning to assess transfer. Subjects also underwent pre- and post-functional magnetic resonance imaging (fMRI) scans while they played the training videogame to assess how these patterns of brain activity change in response to training. For regions implicated in working memory, such as the superior parietal lobe (SPL), individual differences in the post-minus-pre changes in activation predicted performance changes in an untrained working memory task. These findings suggest that training-induced plasticity in the functional representation of a training task may play a role in individual differences in transfer. Our data support and extend previous literature that has examined the association between training related cognitive changes and associated changes in underlying neural networks. We discuss the role of individual differences in brain function in training generalizability and make suggestions for future cognitive training research.

## Introduction

Cognitive neuroscience has begun to explore the possibility of enhancing working memory through the use of videogame-based training products. It has been demonstrated that such videogame training can have a positive impact on the performance of untrained tasks (Green and Bavelier, 2003, 2007; Boot et al., 2008, 2010; Thorell et al., 2009; Van Muijden et al., 2012). A predominant hypothesis of how this occurs is that training affects untrained tasks when they share overlapping cognitive or neural processes with the training (Jonides, 2004; Dahlin et al., 2008b). This extends an older hypothesis in which transfer of training is based on behavioral overlap between trained and untrained tasks (Woodworth and Thorndike, 1901).

Working memory is a cognitive construct that represents the ability to encode, store, and manipulate information in memory (Baddeley, 1992; D'Esposito et al., 1995). Several brain regions in the frontal and parietal cortices and striatum (caudate, putamen) are known to be involved in working memory, including the dorsal lateral pre-frontal cortex (Braver et al., 1997; D'Esposito et al., 2000; Funahashi, 2006), superior parietal lobe (SPL) and precuneus, (Cohen et al., 1997; Henson et al., 2000; Pessoa et al., 2002; Dahlin et al., 2008b; Koenigs et al., 2009), and caudate (Levy et al., 1997; Postle and D'Esposito, 1999, 2003; Bäckman et al., 2011). The involvement of these regions in working memory suggests that individual differences in the function of these regions may also be linked to individual differences in working memory performance (Kane and Engle, 2002). Similarly, previous research has demonstrated that individual differences in the volume of certain brain regions that are important for working memory and procedural learning, such as the striatum, predict learning in complex videogame training (Erickson et al., 2010; Basak et al., 2011).

While previous research has demonstrated working memory training transfers selectively to untrained tasks that share cognitive and neural processes (measured by functional MRI activation) with the training task (Dahlin et al., 2008b), it is undefined how training-induced changes in the neural representation of the training task are related to performance changes in untrained tasks. The neural representation of a task can manifest in a variety of contexts and neuroimaging measurements, but in the current study we use this term to refer to patterns of activation during the training task as measured by an fMRI blood-oxygenation-level dependent (BOLD) contrast between task engagement and quiet rest. As a trainee learns a task or skill, the neural representation of the task changes considerably, both within and between training sessions, including increases and decreases in activation (Garavan et al., 2000; Kelly and Garavan, 2005; Kelly et al., 2006; Dayan and Cohen, 2011). How these changes in the neural representation relate to performance changes in untrained tasks has yet to be examined; however, extending the shared cognitive processing and neural overlap hypothesis, it is reasonable to predict that the plasticity in working memory associated brain regions following working memory videogame training, as measured by changes in brain activation patterns during game play, should relate to changes in the performance of an untrained working memory task.

While it is understood that training can induce changes in task-associated brain activity, it is unclear whether these changes will be an increase or decrease in activation (Buschkuehl et al., 2012); therefore in the current study we remain agnostic to the direction of change in training-associated brain activity. Instead we assert that greater performance changes in a working memory task (Sternberg Memory Search, SMS) should be mirrored by greater plasticity (measured by post-minus-pre brain activity in the training task) in the brain regions associated with working memory. Working memory describes the cognitive processes of storing, manipulating, and updating information in memory (Baddeley, 1992). Similarly, the SMS task taps working memory by asking participants to store and update sets of letters in memory (Sternberg, 1966), and accordingly many other studies have used this task or similar tasks as a measure of the storage and maintenance of information in working memory (Awh et al., 1996; Rypma and D'Esposito, 1999; Raghavachari et al., 2001; Jensen and Tesche, 2002). Previous research has demonstrated that individual differences in working memory performance, assessed independently of MRI scanning, are linked to working memory task-based activation in regions associated with working memory, such as the prefrontal cortex and regions of the parietal cortex (Kane and Engle, 2002; Todd and Marois, 2005). These findings offer further support for the prediction that individual differences in training-induced frontal-parietal plasticity during a working memory oriented training task would relate to individual differences in performance changes in an untrained working memory task.

In the current study, trainees performed two untrained tasks before and after training with Space Fortress for 15 2-h sessions. Before and after training, participants also were scanned using fMRI while playing Space Fortress. To test whether performance changes in an untrained working memory task can be predicted by plasticity in regions associated with working memory, we first correlated pre-training brain activity during game play with changes in performance of two untrained tasks. We performed the same analyses on the post-training brain activity, and these “pre- and post-analyses” served as the basis of our “plasticity analysis,” in which we investigated the relationship between training-induced plasticity and performance changes in untrained tasks. To test whether the association between brain activity during Space Fortress would be related only to untrained tasks that shared cognitive overlap with Space Fortress, we used tasks that were cognitively similar or dissimilar to the processes occurring during the training task.

Space Fortress is an interactive, score based, complex videogame that has a long history of use as a multisensory training tool (Fabiani et al., 1989; Gopher et al., 1989; Donchin, 1995; Kramer et al., 1995, 1999; Vo et al., 2011); it makes high demands of working memory storage and updating, motor control, and attention. The structure of the SMS task has components that are directly related to activities in the Space Fortress training. Specifically, both tasks ask participants to store and update sets of letters for a subsequent response. Furthermore, the response pattern in the SMS task, whether a letter belonged to the most recent letter set, is mirrored directly by Space Fortress in asking participants whether the letter on screen refers to a “friend” or “foe,” which is determined by a letter set given before each Space Fortress trial. Given that Space Fortress engages working memory, and that the SMS task largely mirrors the working memory storage and updating components of Space Fortress, we hypothesize that individual differences in the neural representation of the Space Fortress game will relate to individual differences in performance changes in the SMS task. Furthermore, given that individual differences in the function of regions associated with working memory are likely related to individual differences in working memory performance (Kane and Engle, 2002), we further hypothesize that activity during Space Fortress in regions associated with working memory relates to individual differences in changes in SMS task performance. For our second untrained task, we used the Change Detection (CD) task. This task functions as a control task because while it taps into attention and working memory processes (Pashler, 1988; Rensink, 2002; Baddeley, 2003), the specific cognitive processes in the CD task are quite distinct from that of the Space Fortress game. For example, Space Fortress asks subjects to monitor changes in a symbol at the bottom of the screen, and if they respond when a dollar sign appears twice in a row, they can receive a bonus. This type of CD differs considerably from the CD task in which visual field changes are neither of a predictable type or location. Space Fortress is also a visually simple game, with easily discernable text symbols, and does not require identification of any masked changes; unlike the CD task, which involves both complex real street scenes, with subtly modified scenes, which are separated by a mask. Based on these differences in the both the dorsal and ventral visual attention components of these two tasks, we hypothesize that activity during Space Fortress will not be associated with individual differences in changes to performance in the CD task.

As hypothesized we show that individual differences in functional activation in pre and post fMRI sessions predict individual differences in performance changes to the SMS task; furthermore, we confirmed our hypothesis of no relationship between functional activation in either pre- or post-fMRI sessions and individual differences in performance changes to the CD task. The results of these two tasks taken together suggest that the neural representations of a training task relate more closely to learning in untrained tasks that share higher degrees of cognitive similarity with the training task, which supports previous research showing that training selectively affects untrained tasks with shared cognitive processes and neural overlap (Dahlin et al., 2008b). The results of these analyses gave us a set of regions to use in our subsequent plasticity analysis, in which we investigated the relationship between training-induced plasticity and performance changes in untrained tasks. As we hypothesized, our pre- and post-analyses only found significant results with the SMS task; therefore, we conducted the plasticity analysis on the SMS task and not the CD task.

Of these regions in which pre- and post-analyses identified a significant association with performance changes in the SMS task, we hypothesized that greater plasticity in working memory associated regions would occur in individuals with greater performance gains in the SMS task. Therefore, for our “plasticity analysis” we created spherical regions of interest (ROIs) surrounding the statistical peaks of the group-level maps from the pre- and post-analyses. To measure the plasticity in brain activity in these regions, we extracted the mean percent signal change from these regions, and took a post-minus-pre difference of the game play compared to fixation contrast. We then used a multiple regression model based on the activity differences in these ROIs to predict performance changes in our untrained tasks. Our results support the neural and overlap hypothesis because we show that the post-minus-pre activity differences in regions associated with working memory, such as the SPL, predicted a significant percentage of the variance in performance changes in the SMS task. These findings suggest that changes in a trainee's neural representation of a training task may predict performance changes of untrained tasks that share cognitive or neural processes with training tasks. Furthermore, while some studies have found weakly significant or non-significant training-induced improvements in untrained tasks at the group level, our results demonstrate that analyzing the relationship between brain activity and untrained task performance at the individual level does reveal a significant association between training-induced plasticity and performance in untrained tasks.

## Methods

### Participants

The University institutional review board (IRB) approved this study. We used flyers posted throughout campus as well as online advertisements to recruit participants. To determine eligibility, we asked potential participants to complete a survey about their video game habits, and experimenters determined the participants' eligibility with individual in-person interviews. These in-person interviews addressed the participants' health, as well as a more detailed assessment of their video game habits. All participants (1) played videogames less than 4 h per week, (2) were right handed, (3) were free from psychiatric illness, neurological disease, and metallic implants, (4) had signed an informed consent form, (5) had normal color vision, (6) had a corrected visual acuity of 20/20 or better, and (7) were between the ages of 18 and 30. For our final sample of 45 trainees, there were *N* = 27 females, with a mean age of 21.74 years (*SD* = 5.09) and mean education of 15.71 years (*SD* = 3.27). We also had a minimal-contact control group of 25 participants; however, given that the current study focuses on individual differences in the effect of Space Fortress training, we did not include the control group in our analyses.

### Space fortress

**Figure 1:**
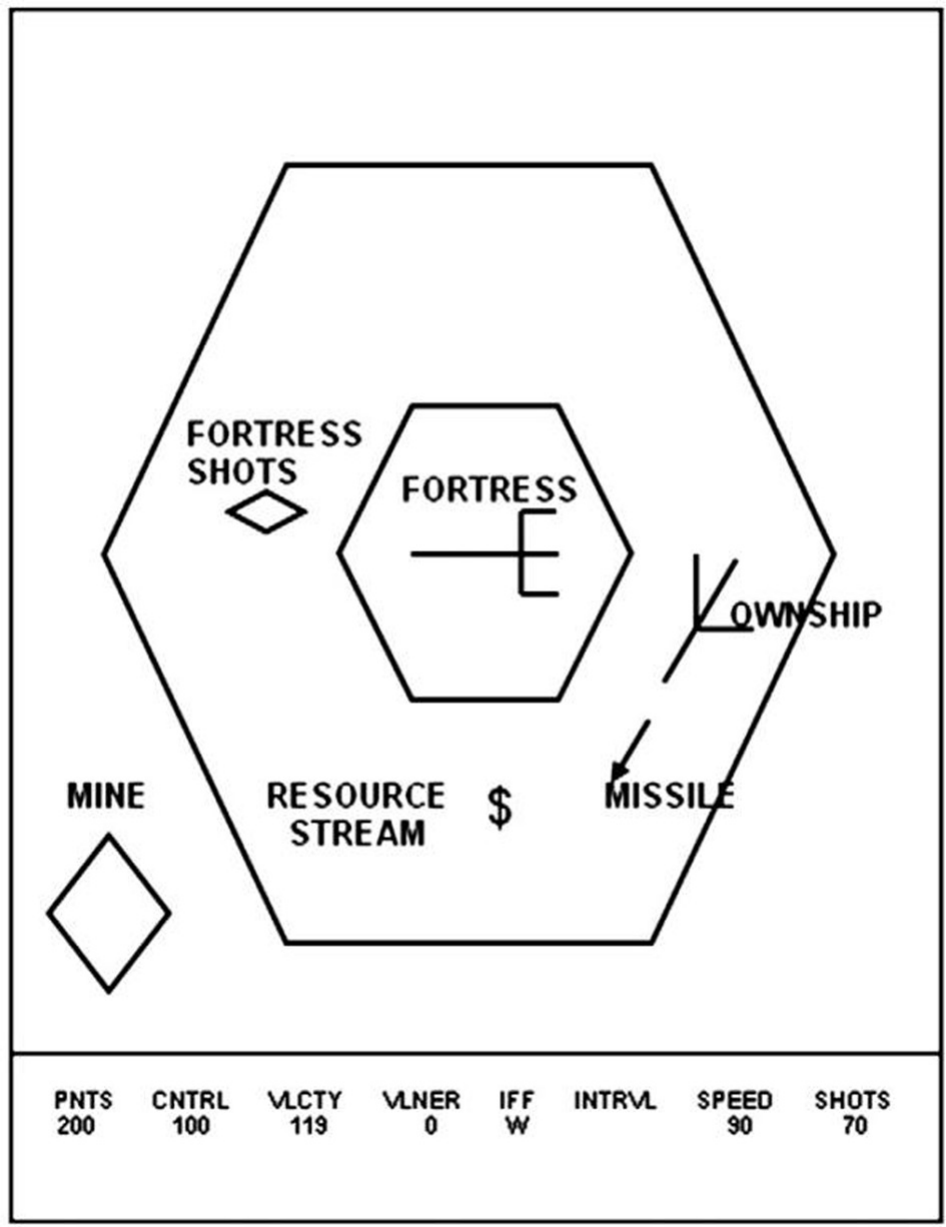
**In this image, a Space Fortress schematic illustrates various components of the game**. The player moves the ship, named “OWNSHIP” in this image, around the screen, while attempting to stay within the surrounding larger hexagon and firing missiles at the central hexagon, which represents the Space Fortress. Mines, bonuses, and other items come across the screen, to which the player must handle quickly and efficiently. The bottom gives indications to the player of their Points score, Control score, Velocity score, as well as the Space Fortress' vulnerability level, the identity of the mine on screen, the mine identification interval (not depicted), the speed score, and the number of shots the player has remaining. This image was taken from previous work using Space Fortress (Lee et al., 2012).

Space Fortress was developed to study the effect of different training strategies on learning, retention and transfer within the context of a rich and cognitively complex task (Figure 1). Playing Space Fortress requires complex motor skills, procedural learning, and working memory. The game score is compartmentalized into four subcategories measuring: (1) points: successfully destroying the space fortress with 10 successive missiles spaced 250 ms apart followed by rapid double shots spaced less than 250 ms; (2) velocity: keeping the ship's movement within a predefined speed range; (3) control: moving the ship only within a predefined allowable area in a frictionless environment without braking; (4) speed: handling friendly and enemy mines quickly and precisely. In addition to these tasks, the participant must maintain three letters in working memory that identify mines as friend or foe. Furthermore, the participant must monitor a stream of symbols that will occasionally present two dollar signs ($) in direct succession, which is an indication of a bonus for the player. For a more detailed explanation of Space Fortress, see Mané and Donchin (1989) or Lee et al. (2012).

#### Training procedure

All participants watched a 20-min instructional video that explained the details of the Space Fortress game, followed by a 5-min summary video and six 3-min games to practice before entering a 3T Siemens Trio scanner. Over the next several weeks, the trainees played the game for a total of 30 h, split into 15 2-h training sessions. Following training, all participants were scanned again with the pre-training protocol.

### Neuropsychological assessment battery

The participants performed pre and post training neuropsychological tasks (i.e., untrained tasks) in three general categories: visual-attention, memory, and multimodal task performance, to measure baseline cognitive abilities and changes of performance as a result of extended Space Fortress practice. These tests have been previously described in detail (Lee et al., 2012). In the current study, we focus on two tasks including the SMS task and a CD task. We focus on the SMS task because this task closely mirrors distinct cognitive components of the Space Fortress training. For example, the SMS task requires subjects to consolidate and maintain visual information within working memory in a fashion similar to Space Fortress. We focus on a CD task because similarly to the SMS task it is thought to tap into attention and the working memory (Pashler, 1988; Rensink, 2002; Baddeley, 2003). However, it is important to note that the stimulus-response processes involved and the contents to be remembered in this task (scenes) are quite distinct from those in the Space Fortress training and SMS task (letters and symbols).

#### Sternberg memory search task

In the SMS task, participants viewed a set of 3 or 5 letters (duration: 1200 ms) followed by a pause (1500 ms), and then a brief presentation of a letter (Sternberg, 1966). Participants needed to respond as quickly and accurately as possible whether this letter belonged to the previously viewed set of letters. Our participants received accuracy feedback for 32 practice trials before being tested on 96 trials without feedback. The SMS task uses reaction times and accuracy as outcome variables. We used accuracy alone to measure performance, because unlike the SMS task, during Space Fortress, there is a delay before the stimulus can be flagged as friend or foe, therefore subjects are encouraged to respond accurately rather than quickly. Conversely, each trial in Space Fortress lasts, on average, longer than each trial of the SMS task, making larger demands on working memory maintenance and therefore accuracy of stimulus-recognition. Performance was measured by averaging accuracy scores in the 3 and 5 letter set conditions. The SMS task taps the storage and maintenance of information in working memory because participants are asked to store letter sets in memory over a delay period, and update this letter set in each trial (Sternberg, 1966).

#### Change detection

In a single trial the participants viewed a repeating cycle of four images: a street scene (240 ms), a gray interruption image (80 ms), a modified version of the original street scene (240 ms), and then another gray interruption image (80 ms), after which the cycle repeated. We asked participants to detect and report a difference between the original and modified image. If they did not detect the difference after 60 s of repeated cycling through the screens, they continued onto the next trial, for a total of 24 trials. We assessed CD accuracy by determining the percentage of correct trials out of all trials that contained a modified image (22 trials).

### MRI data acquisition

In the MRI sessions, we collected an MPRAGE T1-weighted high-resolution structural volume with 144 contiguous axial slices, collected in an ascending fashion and parallel to the anterior posterior commissure line (160 × 192 × 144 voxels, voxel size 1.33 × 1.33 × 1.30 mm, echo time (*TE*) = 3.87 ms, repetition time (*TR*) = 1800 ms, field of view (*FOV*) = 256 mm). Then, for the Space Fortress scans, we acquired three runs of T2^*^ weighted EPI images for BOLD signal acquisition (*TE* = 25 ms, *TR* = 2 s, Flip angle 80°, voxel size 3.475 × 3.475 × 4 mm, 28 slices, 64 × 64 voxels matrix, BOLD volumes in each functional scan = 115). While in the scanner, the participants alternated between 30-s blocks of fixating on a central cross (Fixation), passively viewing a recorded session of an experienced player's Space Fortress session, and playing the full Space Fortress game (Space Fortress). We began with a sample size of 50 trainees, and we excluded participants based on excessive motion artifacts. All images were collected on a 3T Siemens TRIO MRI scanner.

#### MRI pre-processing and analysis

**Figure 2:**
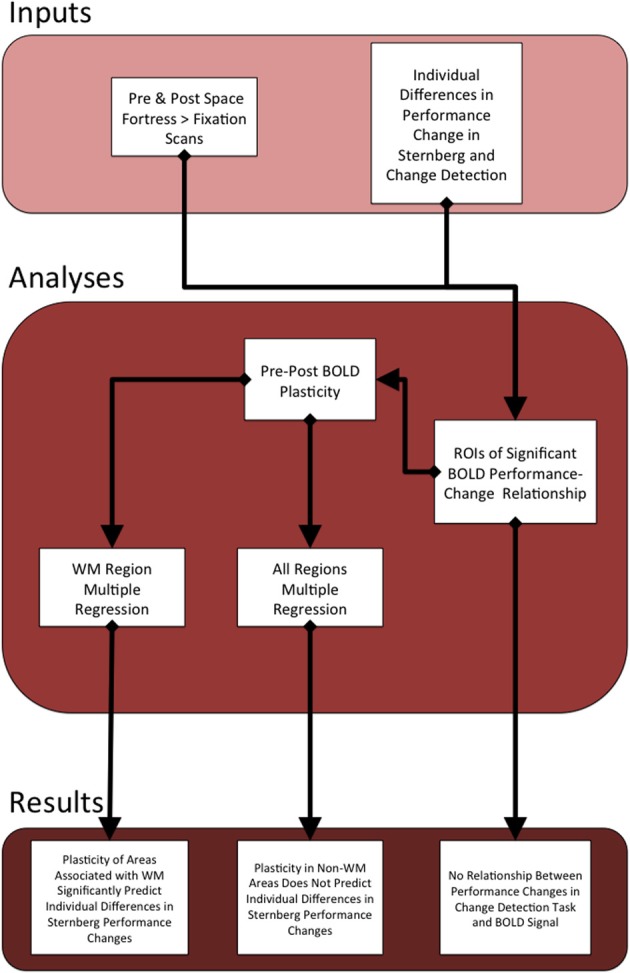
**This figure serves as an outline of the current study**. The top section describes the behavioral and fMRI inputs that we used to find statistical peaks in the relationship between Space Fortress BOLD signal and the individual differences in changes in performance to both the SMS task and CD task. We calculated separate pre- and post-analyses for both the SMS and CD behavioral data. Given that the BOLD signal only showed a relationship to the SMS task, we only created ROIs surrounding the statistical peaks in the SMS task fMRI analysis. We then extracted the percent signal change from all of these ROIs from both pre- and post-sessions, and subtracted the percent signal change of pre from post to obtain our metric of the change in neural representation of the Space Fortress task, which we operationalize as brain plasticity. Then, we applied the metrics of plasticity from the regions associated with working memory to a multiple regression analysis, to predict individual differences in performance changes in the SMS task. The plasticity values from all regions were entered into a separate multiple regression equation to assess whether regions not associated with working memory would supply a unique contributtion to the variance in individual differences in performance changes to the Sternberg task, which was not the case.

All pre-processing and subsequent analyses of the MRI data were performed using FSL (FMRIB Software Library) (Smith et al., 2004; Woolrich et al., 2009; Jenkinson et al., 2012). We applied rigid body motion correction using MCFLIRT (Jenkinson et al., 2002), and then used BET to remove non-brain structures (Smith, 2002). We applied spatial smoothing using a Gaussian kernel with an 8.0 mm full width half maximum and applied a temporal high-pass filter of 220 s to remove low-frequency signal of non-interest. For the individual-level analyses of each participant, the hemodynamic response was modeled and convolved with a double gamma function in each of the three individual-level runs. Each of the three runs was registered linearly to the subject's MPRAGE using FLIRT (Jenkinson and Smith, 2001; Jenkinson et al., 2002). Then, individual-level statistical maps were forwarded to a fixed effects analysis, and these results were linearly registered to the standardized 2 mm ICBM-152 Montreal Neurological Institute (MNI) Template (Mazziotta et al., 2001). See Figure 2 for flow chart of the current study's analyses and results.

### Associating the space fortress bold signal with changes in untrained task performance

It is likely that the BOLD signal in the Space Fortress > Fixation contrast is more informative of individual differences in working memory processing in the context of a complex task, compared to a Passive > Fixation contrast. Similarly, we assert that the Space Fortress > Fixation contrast is more informative than a Space Fortress > Passive contrast for the reason that in the Passive condition the participants may still engage in working memory processes, which we are interested in investigating in our study. Therefore, contrasting Space Fortress with Passive viewing may remove such working memory-associated activity of interest. Because of these reasons, we chose to focus on the Space Fortress > Fixation contrast in our analyses, and therefore, all mentions of brain activity and refer to this contrast.

For our higher-level analysis, the individual level of fixed effect images of the Space Fortress > Fixation contrast were submitted to a mixed effects group analysis in FSL's FEAT (Worsley, 2001). We performed these analyses for two reasons, first to investigate whether individual differences in brain activity before or after training relate to individual differences in gains in untrained tasks, and second, to find a targeted set of areas to use in our subsequent plasticity analyses of whether individual differences in training-induced plasticity relate to individual differences in performance change in untrained tasks.

**Figure 3:**
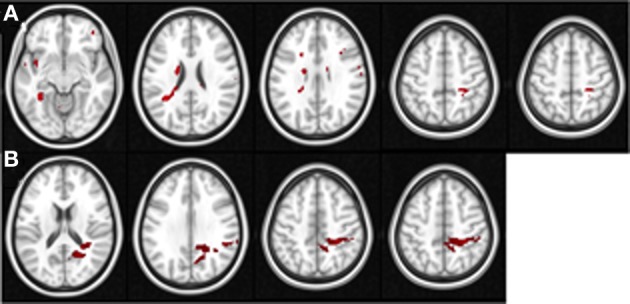
**These figures display the location of the areas in the pre-training (A) and post-training sessions (B) in which the Space Fortress > Fixation BOLD signal demonstrates a significant correlation with individual differences in performance changes in the SMS task**. The axial slices are arranged in ascending order, and the z coordinate value in MNI space is placed above each slice. Each axial slice contains at least one of the peak ROIs of this analysis. All peaks are shown, and for viewing purposes, the statistical maps are set to a *Z* threshold of 3.0 in **(A)**, and 2.3 in **(B)**.

In order to examine the correlation between the BOLD signal and changes in performance in the untrained tasks, we used the performance change scores (i.e., the tasks performed before vs. after the completion of Space Fortress training) from the SMS and CD tasks as regression covariates in two separate group analysis design matrices for both the pre and post fMRI scan, yielding four total group analyses in total. We used a Z-statistic threshold of 1.96 and cluster *p*-value threshold of 0.01 for our mixed effects statistical maps. This *Z* threshold would be considered low for a standard GLM contrast; however, since in this analysis we correlated BOLD contrast with a behavioral variable, we believe that a *Z* threshold of 1.96 (two tailed Type I error rate of 0.05) and a cluster *p*-value threshold of 0.01 are reasonable. This analysis yields a statistical brain map of *Z* scores that reflect the strength of the association between individual differences in changes in untrained performance and the Space Fortress > Fixation contrasted BOLD signal, and we performed this mixed effects analysis in both pre- and post-fMRI scans separately (Table 1; Figure 3). The Harvard-Oxford Cortical Structural Atlas and Harvard-Oxford Subcortical Structural Atlas are the probabilistic atlases in FSL that defined our location labels of our ROIs.

**Table 1:** **This table summarizes the locations of the fMRI activation predictors in the current study**.

**STERNBERG MEMORY SEARCH TASK**			
	**MNI Coordinates**	**Peak *Z*-Value**	**Space Fortress Pearson's *r***
**PRE-TRAINING**			
Caudate-L	−18, −8, 24	4.12	0.523
Fusiform cortex-L	−34, −46, −10	4.11	0.522
Insular cortex-L	−40, 4, −10	3.70	0.483
PCG-R	62, −14, 28	3.16	0.426
PCG SPL-R	28, −36, 52	3.98	0.510
SPL-R	26, −44, 50	3.24	0.435
Supramarginal gyrus-R	64, −20, 24	3.04	0.413
**POST-TRAINING**			
PCG-R	18, −36, 48	2.84	0.390
Precuneus-R	22, −56, 18	3.28	0.439
SPL-R	28, −38, 46	3.35	0.447
Supramarginal gyrus-R	50, −40, 30	2.81	0.386
**CHANGE DETECTION TASK**			
	**MNI coordinates**	**Space Fortress *Z*-Value**	**Space Fortress Pearson's *r***
**PRE-TRAINING**			
No areas passed a threshold of *Z* = 1.96			
**POST-TRAINING**			
No areas passed a threshold of *Z* = 1.96			

The top portion of the table corresponds to ROIs that demonstrated a significant relationship between pre-training BOLD activity and performance changes to the SMS task, while the middle of this table corresponds to ROIs that demonstrated a significant relationship between post-training BOLD activity and performance changes to the SMS task. The pre-training BOLD activity peaks all belong to a single 15,725 voxel cluster, and the post-training BOLD activity peaks all belong to a single 1896 voxel cluster. The bottom portion of the table shows the null results found in the CD task. In the first column, the names of the approximate regions in which the ROIs are located, as according to the Harvard-Oxford Cortical and Subcortical Structural Atlases. Each ROI name is accompanied by an L to denote that it belongs to the left hemisphere, or an R to denote that it belongs to the right hemisphere. In the second column, MNI coordinates of the same ROIs are provided. The third column provides the significance level of the relationship between transfer and the BOLD signal for the Space Fortress > Fixation contrast at that given ROI. All ROIs in this column passed a Z = 1.96 significance threshold and a p = 0.01 cluster threshold. The fourth column offers a reverse of the Fisher's Z transformation to offer an averaged Pearson's r for all voxels contained in each 10 mm peak ROI. These r-values were calculated based on the following formula: *r* = $\frac{Z^{2}}{Z^{2}+N}$, where Z is the significance value, and N corresponds to sample size.

### Multiple regression analysis of brain plasticity

In order to investigate the effects of brain plasticity in Space Fortress on changes to SMS task performance, we created spherical ROIs, (10 mm in diameter and 264 mm^3^ in volume), surrounding the peak *Z*-statistic values from the pre- and post-mixed effects analyses. Since the pre- and post-mixed effects analyses looks for regions of the brain that have significant association with performance changes in the untrained tasks, we hypothesized that any generalized learning that occurs during Space Fortress training would manifest in brain plasticity in these same regions. Given that this analysis was based on these statistical peaks, the null mixed-effects results for the CD task prevented us from including the CD task in the multiple regression analyses. We extracted percent signal change (%SC) from these ROIs from both sessions' fMRI scan and took the post-minus-pre difference. This step allowed us to restrict our search to areas of interest that were related to performance changes in the SMS task, while not causing problems of statistical resampling because we created new metrics of plasticity in these ROIs by creating a post-minus-pre training BOLD contrast, rather than using the brain activity from either session alone. By entering these values into a multiple regression we were able to assess the percentage of variance in changes in SMS task performance that are accounted for by changes in brain activation during performance of the Space Fortress game.

We used SPSS to calculate a backward multiple regression, in which the full model of all variables is considered, and then each variable is iteratively removed and the significance of the model is reassessed. In all iterations, the variable that contributes the least variance to the model is removed until each remaining variable contributes significant variance to the regression. This method results in a regression model in which each independent variable predicts a significant percentage of the variance in the dependent variable, but unlike the stepwise multiple regression model, the backwards model is not biased by the order in which the variables are added to the model, since all are considered initially. Given that the working memory component of Space Fortress may contribute to changes in processing in the SMS task, our first multiple regression used plasticity values only from brain regions that have been shown to be involved in working memory: the SPL, caudate, precuneus, and postcentral gyrus (PCG) (Cohen et al., 1997; Levy et al., 1997; Henson et al., 2000; Pessoa et al., 2002; Dahlin et al., 2008a,b; Koenigs et al., 2009; Bäckman et al., 2011); we used backwards multiple regression to create a model from the plasticity in these regions. Since this analysis included only a restricted set of ROIs, a counter-hypothesis would be that any plasticity, regardless of brain region, could have significantly predicted performance changes to the Sternberg task. To confirm our hypothesis that the plasticity in working memory regions specifically accounted for the variance in performance changes to the SMS task, we compared the regression model of working memory regions alone, to a larger model, in which we added the remaining regions that were deemed significant in the mixed effects analysis: the supramarginal gyrus, temporal fusiform cortex, and the insular cortex.

## Results

### Performance changes on untrained tasks after space fortress training

On average, participants did not demonstrate significantly different pre-to-post scores in the SMS task [pre-training mean 94.4%; 95% *CI* = 93.2–95.6%; post-training mean 93.5%; 95% *CI* = 91.6–95.3%; paired *t*-test *t*_(44)_ = 1.32, *p* > 0.05]. We also did not find significant pre-to-post differences in the CD task [pre-training mean 85.3%; 95% *CI* = 82.3–88.3%; post-training mean 86.8%; 95% *CI* = 83.9–89.8%; paired *t*-test, *t*_(44)_ = 0.935, *p* > 0.05]. We did not find a reaction-time accuracy trade off from pre- to post-session for either of the tasks. The current study focuses on individual differences in performance changes of these tasks.

### Neural representation of space fortress videogame predicts individual differences in changes to performance in an untrained task

In our pre- and post-analyses we investigated the relationship between BOLD activity during Space Fortress performance and individual differences in performance changes in untrained tasks (SMS and CD tasks). We did so by using performance change scores as covariates of interest in a between-subjects analysis for both pre- and post-sessions separately (Table 1). We found that pre- and post-session BOLD signal in several frontal, parietal, and subcortical regions demonstrated a significant association with performance changes in the SMS task, but not in the CD task (Table 1). During the pre-training fMRI session, activity in the caudate, PCG, and SPL was positively associated to individual differences in performance changes in the SMS task (Figure 3A). In the post-training fMRI session, activity in the SPL, precuneus, and PCG were positively associated with performance changes in the SMS task (Figure 3B). These results corroborate our hypothesis that patterns of brain activation obtained during the performance of the Space Fortress task would be associated with individual differences in performance change in the SMS task and that these relationships would be manifested in regions of the brain known to be involved in working memory, such as the SPL, caudate, precuneus. These results also support our hypothesis that this relationship would exist for performance changes in untrained tasks sharing cognitive processes with the training and not those using dissimilar cognitive processes. Since we did not find any brain regions with a significant association between signal to performance changes in the CD task, and the ROIs for our regression analyses were created by extracting the data surrounding the statistical peak activations in the mixed effects analysis, we could not include the CD task in the multiple regression analyses.

### Frontal-parietal brain plasticity predicts individual differences in improvements in an untrained working memory task

In our plasticity analysis we investigated whether changes in the neural representation of Space Fortress predicted a significant percentage of the variance in performance changes in the SMS task. First, we included the mixed effects derived ROIs in the SPL, caudate, precuneus, and PCG, which have all been associated with working memory (Cohen et al., 1997; Levy et al., 1997; Postle and D'Esposito, 1999, 2003; Henson et al., 2000; Pessoa et al., 2002; Dahlin et al., 2008b; Koenigs et al., 2009; Bäckman et al., 2011). We found that using backwards multiple regression, changes in %SC predicted 32% of the variance in the performance changes to the SMS task [Working Memory Model-*R*^2^: 0.37; adjusted *R*^2^: 0.32; *F*_(44)_ = 8.040, *p* < 0.01] (Table 2). Adjusted *R*^2^ is an estimate of how well the same model would perform in an independent sample taken from the same population. These results support the notion that plasticity in regions important for working memory would have an impact on working memory processes of similar tasks. Greater decreases in activity in the SPL and PCG (Standardized Beta = −0.347, and −0.264, respectively), and greater increases in activity in the precuneus (Standardized Beta = 0.392) were associated with greater improvements to the SMS task. These standardized beta values indicate the importance of each variable in the model. Therefore, the increases in activity in the precuneus, and decreases in the SPL and PCG contribute to our model's significant prediction in declining order of importance.

**Table 2:** **This table summarizes the progression of the backwards multiple regression model from including the plasticity of several regions associated with working memory to including only the Precuneus, PCG SPL, and PCG**.

**Model Summary**				
**Model**	***R***	***R***^2^****	**Adjusted *R***^2^****	**Std. error of the estimate**
1	0.614^a^	0.377	0.259	0.084
2	0.614^b^	0.377	0.279	0.082
3	0.614^c^	0.377	0.297	0.081
4	0.613^d^	0.376	0.314	0.080
5	0.609^e^	0.370	0.324	0.080

In backwards multiple regression, the first model begins with all available information, and in each round removes the least important variable until it converges on an optimal solution. This iterative model attempts to maximize the adjusted R^*2*^-value, which is an estimate of the explanatory power of this model in an independent sample drawn from the same population.

a Predictors: (Constant), SPL (32, −42, 62), SPL (26, −44, 50), Caudate (−18, −8, 24), Precuneus (22, −56, 18), PCG SPL (28, −36, 52), PCG (18, −36, 48), PCG (62, −14, 28).

b Predictors: (Constant), SPL (32, −42, 62), SPL (26, −44, 50), Precuneus (22, −56, 18), PCG SPL (28, −36, 52), PCG (18, −36, 48), PCG (62, −14, 28).

c Predictors: (Constant), SPL (32, −42, 62), SPL (26, −44, 50), Precuneus (22, −56, 18), PCG SPL (28, −36, 52), PCG (62, −14, 28).

d Predictors: (Constant), SPL (32, −42, 62), Precuneus (22, −56, 18), PCG SPL (28, −36, 52), PCG (62, −14, 28).

e Predictors: (Constant), Precuneus (22, −56, 18), PCG SPL (28, −36, 52), PCG (62, −14, 28).

**Figure 4:**
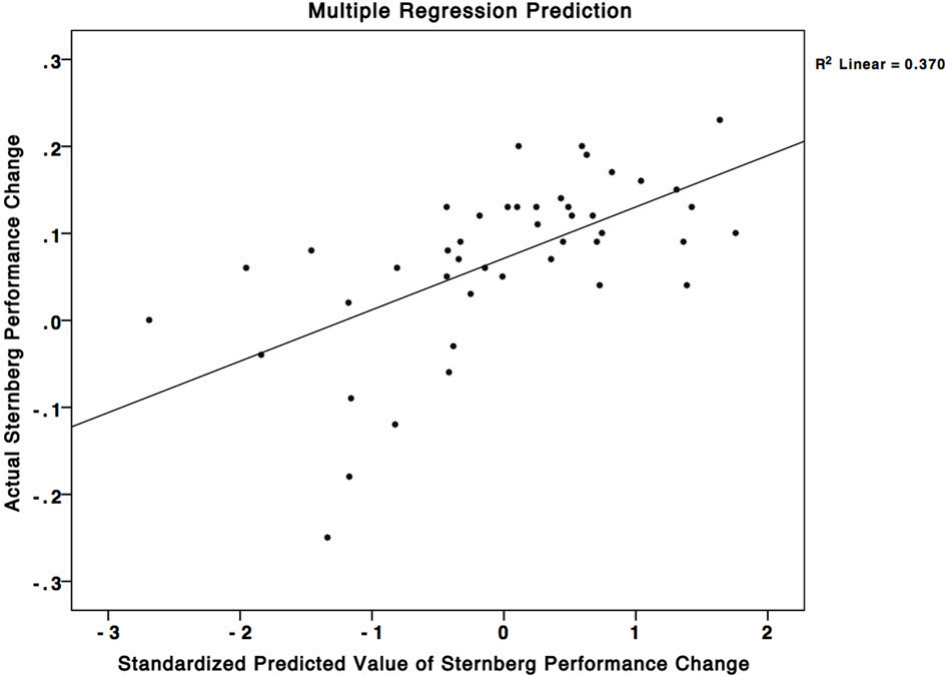
**A multiple regression equation using changes in brain activity in the SPL, PCG, and precuneus was able to predict 37% of the variance in performance changes in the SMS task**. This scatterplot graphs this relationship. The X-axis corresponds to standardized predicted performance changes in the SMS task using the model based on change in brain activity, while the Y-axis corresponds to the actual changes in the SMS task. The squared correlation between the X and Y axes corresponds to an *R*^2^-value of 0.37.

To test our hypothesis that the plasticity in working memory regions alone would account for the variance in performance changes to the SMS task we used all the ROIs from the mixed effects analyses in a larger multiple regression analysis. We added post-minus-pre activity change scores from ROIs in the SPL, caudate, PCG, precuneus, supramarginal gyrus, temporal fusiform cortex, and the insular cortex to the analysis. We found a non-significant 3% *R*^2^ improvement of the regression model from 37% (Figure 4) (working memory associated regions only) to 40% (all regions) (*F* = 1.300, *p* > 0.05). These results suggest that individual differences in activity-changes in working memory associated areas may be particularly important for predicting individual differences in performance changes in similar working memory tasks, and that plasticity in regions that have not been shown to be involved in working memory may not contribute to performance changes in such tasks. This result is important because it gives an insight into how individual differences in plasticity that occur during training can determine how the trainees change their performance on untrained tasks.

## Discussion

### Implications of the current study

The findings of our plasticity analysis demonstrate that changes in BOLD signal in the SPL, PCG, and precuneus, from pre- to post-training using a videogame with a working memory component, predict changes in performance in an untrained working memory task. Previous research has simultaneously found significant changes in activation in working memory associated regions, such as the SPL and caudate, in response to working memory training along with improvements in an untrained working memory task (Dahlin et al., 2008b). Our findings extend this research by demonstrating that the changes in functional activation that occur during working memory training predict individual differences in changes in untrained working memory task performance. These findings suggest that as the functional processing of Space Fortress changes following training, so does the functional processing of the SMS task, which supports previous notions that the training-induced plasticity in brain regions associated with training and untrained tasks is associated with transfer to untrained tasks (Jonides, 2004; Dahlin et al., 2008b). These findings also confirm hypotheses of others suggesting that the frontal-parietal network serves as a basis for transfer between working memory tasks (Klingberg, 2010).

In our pre and post analyses we also found a distributed set of brain regions in which the Space Fortress > Fixation BOLD signal at either pre or post fMRI scan correlated with performance changes in a working memory task. This analysis included regions that have been associated with working memory in previous research, such as the caudate (Levy et al., 1997; Postle and D'Esposito, 1999, 2003; Bäckman et al., 2011) and SPL (Cohen et al., 1997; Henson et al., 2000; Pessoa et al., 2002; Dahlin et al., 2008b; Koenigs et al., 2009). Given that previous literature has demonstrated that these regions play an important role in working memory, our findings suggest that these regions may also play a role in the relationship between training in a complex videogame, such as Space Fortress, and individual differences in performance changes in a working memory task.

Counter to our hypotheses, activation in brain regions aside from those associated with working memory and updating, such as the temporal-occipital fusiform cortex, were also associated with performance changes in an untrained working memory task. One interpretation of these findings is that the relationship between brain activity during Space Fortress and the untrained working memory task performance change is non-specific to regions associated with working memory. However, in follow-up analyses the multiple regression model that included these additional regions showed no improvement in model performance compared to the working memory model. This aids our interpretation of the results by indicating that the relationship between activity during Space Fortress and changes in performance in a working memory task are specifically explained by changes in activity in regions associated with working memory.

Space Fortress is a complex task, and it makes demands on working memory, motor control, and attention. While single components of the task are related to other cognitive processes, the training as a whole is different and more difficult than many individual cognitive tasks. In light of the multimodal nature of the training, one may expect that the learning that occurs during training would only be represented by performance changes in tasks similar to the whole training task. However, given that we found relationships between Space Fortress brain activity and changes in performance to the cognitively similar SMS task but not the cognitively dissimilar CD task, our results suggest that training-component similarity may be sufficient for the training to affect untrained tasks. Furthermore, this similarity may be important for assessing how individual differences in brain plasticity predict changes in performance in untrained tasks, which agrees with previous notions that cognitive overlap between training and untrained tasks is critical in predicting transfer of training (Jonides, 2004; Dahlin et al., 2008b). We interpret these results as suggesting that training on a complex task induces a change in the representation of the training task across a variety of functional brain networks, and that these changes may affect a group of untrained tasks that are limited to those tasks which are functionally represented in a similar manner as a component of the complex training task that induces significant brain plasticity. This interpretation is supported by previous literature suggesting that training should preferentially affect those tasks that share elements of neural (Dahlin et al., 2008a,b) or behavioral similarity (Woodworth and Thorndike, 1901).

### Limitations

While our findings offer several suggestions that are in line with previous cognitive training literature, they should be interpreted with some limitations in mind. First, it is well known that a properly controlled cognitive training experiment should include a group which trains with a control training task that is selected or created to minimize the difference in expectancy effects between the training and the control group (Boot et al., 2011). In other words, all participants should be blind to whether they belong to an experimental or control group, which is thought to minimize the effect of their own expectations on their training outcome. This is quite difficult to achieve in most laboratory settings. Nevertheless, to investigate questions of the efficacy of specific training components, some researchers have used modified high and low interference versions of a working memory task as experimental and active control conditions, respectively (Oelhafen et al., 2013). Our study does not include such an active control group with the removal of a single training component. Therefore, we cannot make strong conclusions on the specific effect of the working memory component of Space Fortress training on our untrained working memory task. Furthermore, we cannot make claims that Space Fortress uniquely had such an effect, as compared to a similarly complex multimodal training task. Thus, our hypotheses concern directly how variation in an individual's representation of the training, recorded by fMRI here but which could also be assessed with sophisticated behavioral metrics such as eye-tracking, predict the trainability of those individuals. We believe our findings provide a good beginning toward the understanding of the inter-relationships between transfer and individual differences in the representation of the training task, which may help future studies assess how to guide this dynamic relationship with the training task to increase the transfer of training. While our findings agree with what previous findings and theory would suggest, future efforts should aim to include such targeted control groups to account for this effect.

### Implications of current findings on questions for future research

The current study offers a variety of practical suggestions for future cognitive training studies. First, while a complex training task may have an effect on simpler untrained tasks that share little relationship with the training task in its entirety, the training task is more likely to have an effect on simpler untrained tasks that share cognitive processes with one or more components of the training task. Second, individual differences in the neural representation as well as changes in the neural representation of a training task can be predictive of how the training will affect untrained tasks. These findings suggest that such individual differences may play a critical role in the outcome of a cognitive training regimen. We suggest that future neuroimaging and cognitive training studies also perform assessments of participant motivation toward training, engagement in the task, and personality metrics, all of which may contribute to pre-training trait-like individual differences in how an individual will benefit from a cognitive training regimen. This information will help answer questions of how individual differences in cognitive training-based plasticity are related to individual differences in motivation, personality, and cognitive ability. Understanding these questions will not only allow for improvements in the development of cognitive training programs, but it will also help future researchers explore the topic of transfer with more clarity. Furthermore, in light of the importance of these individual differences, our findings support the suggestions of others that “one-size-fits-all” training regimens may be inappropriate, and training paradigms that cater to individual differences in trainability or other personal attributes may improve the effect of the cognitive training regimen (Jaeggi et al., 2011; Buschkuehl et al., 2012).

While the current study offers insight on how brain plasticity contributes to performance changes in untrained tasks, many questions remain. For example, one important future issue will be elucidating the difference in short-term vs. long-term brain plasticity that occurs within vs. between training sessions. By making these differences more clear, future cognitive training studies could investigate whether short-term and long-term learning uniquely contribute to performance changes in training tasks, or whether individual differences in these short-term and long-term learning lead to individual differences in generalizability of the training.

Finally, we suggest that future research focus on maximizing training-induced brain plasticity by combining cognitive training with other interventions that are thought to encourage states of plasticity, such as exercise, which increases many plasticity associated biomarkers, such as brain-derived neurotrophic factor (Cotman et al., 2007; Hillman et al., 2008; Van Praag, 2009), a protein that is thought to be important for the growth and differentiation of new neurons and synapses (Cotman et al., 2007). Trans-cranial current stimulation is another intervention technique that is thought to encourage neuroplasticity by raising levels of several biochemical markers of plasticity, including myoinositol (Hunter et al., 2013), which is associated with the long-term potentiation second messenger system, which is important for the growth of synapses (Rango et al., 2008). Given the relationship we found between plasticity in Space Fortress training and performance changes in an untrained task, we suggest combining these other intervention techniques with cognitive training may increase training-induced plasticity thereby increasing the transfer of training.

### Conflict of interest statement

The authors declare that the research was conducted in the absence of any commercial or financial relationships that could be construed as a potential conflict of interest.
